# Development of a novel and cost-effective redox sensor for voltammetric determination of pantoprazole sodium during pharmacokinetic studies

**DOI:** 10.1098/rsos.170324

**Published:** 2017-08-09

**Authors:** Pakinaz Y. Khashaba, Hassan Refat H. Ali, Mohamed M. El-wekil

**Affiliations:** 1 Department of Pharmaceutical Analytical Chemistry, Faculty of Pharmacy, Assiut University, Assiut, Egypt; 2 Department of Pharmaceutical Analytical Chemistry, Faculty of Pharmacy, Deraya University, El-Minya, Egypt

**Keywords:** pantoprazole sodium, pretreated pencil graphite electrode, pharmacokinetic study, square wave adsorptive stripping voltammetric

## Abstract

A pencil graphite electrode modified with poly (bromocresol green (BCG)) was prepared by electro-polymerization process for the determination of pantoprazole sodium. The surface morphology and structure of poly (BCG) film were characterized by scanning electron microscopy and Fourier transform infrared spectroscopy. The determination of pantoprazole sodium in Britton–Robinson buffer (pH 7.0) was carried out by square wave adsorptive stripping voltammetric technique. Under optimum conditions, the linear response of the peak with concentration of the cited drug was in the range of 6.6–360 × 10^−8 ^M with limit of detection of 2.2 × 10^−8 ^M. Moreover, the poly (BCG)-modified electrode has been successfully applied to determine pantoprazole sodium in tablets, vials and during pharmacokinetic studies.

## 1. Introduction

Pantoprazole sodium (PAN sodium) is a member of the benzimidazole class of proton pump inhibitors. It is commonly used in the management of gastrointestinal ulcers and reflux esophagitis [1]. Its efficacy as antiulcer and anti-secretory agent has been well established. Several methods have been previously used for its determination in pharmaceutical dosage forms and biological fluids including UV-visible spectrophotometry [2,3], spectrofluorimetry [4], high-performance thin-layer chromatography [5], high-performance liquid chromatography [6,7], capillary electrophoresis [8,9] and electrochemical methods [10–16]. The previous electrochemical methods for the determination of PAN sodium had relied on using glassy carbon, carbon paste and *ex situ* plated antimony-film glassy carbon electrodes. All of these approaches require some degree of expertise, preparation and care. Voltammetry has many outstanding features, including high sensitivity, accuracy and ease of operational procedures. Owing to their low background current, wide potential window, chemical inertness and low cost, several forms of carbon electrodes are available for electro-analytical applications [17–19]. Among these, the pencil graphite electrode (PGE) has been applied to voltammetric detection of trace metals [20,21], DNA [22,23], immunoassay [24] and some drugs in pharmaceutical formulations [25]. As the thickness, permeation and charge transport of polymeric films can be controlled by adjusting the electrochemical parameters, polymer-modified electrodes have the advantages of improving electro-catalysis, the absence of surface fouling and prevention of undesirable reactions competing kinetically with the desired electrode process [26–28]. It has been demonstrated that polymer-modified electrodes, especially those coated with dyes and dyestuffs show excellent stability, reproducibility and homogeneity [29–31]. Most of the redox dyes are artificial electron donors [32,33], and they are able to undergo electro-polymerization. Polymerization of bromocresol green (BCG) as a redox mediator on the surface of PGE has the following advantages [34]: (i) has potential for incorporation of counter ions or other functionalities against analyte of interest; (ii) comprises electronic properties similar to metals in addition to conventional properties of organic polymers; (iii) interferences can be avoided by formation of very selective coatings which can distinguish between analyte of interest and other species via hydrophobic and hydrophilic affinities, electrostatic interaction or ion-exchange abilities; and (iv) they help in avoiding electrode poisoning or fouling of the bare electrode by forming a protective surface. Recently, few papers used poly (BCG) for analysis of serotonin after modification with Fe_3_O_4_ in chitosan matrix [35]; glutathione after modification with multiwalled carbon nanotubes [36]; and mixture of ascorbic acid, uric acid and dopamine using glassy carbon electrode [37].

In this study, BCG was chosen as a monomer to obtain a film of poly (BCG) on PGE by electrochemical polymerization for, to our knowledge, the first time. Also, because of high electron density of hydroxyl in the BCG molecule, the BCG film has high concentrations of negatively charged surface-functional groups. The developed poly (BCG)/PGE has been applied to determine PAN sodium electrochemically in pharmaceutical formulations with *in vivo* and pharmacokinetic studies.

## 2. Experimental procedure

### 2.1. Standard materials

PAN sodium was supplied as a gift from Sigma, Quesna, El-Menoufia, Egypt. Domperidone was supplied as a gift from EIPICO, El-Sharqia, Egypt. Aceclofenac, tinidazole, clarithromycin were obtained as gifts from NODCAR, El-Giza, Egypt. Doxycycline was supplied as a gift from CID, Assiut, Egypt. Pantoloc^®^ tablets (MUP, Cairo, Egypt), labelled to contain 40 mg PAN. Pantazol^®^ vials (Sigma, Quesna, El-Menoufia, Egypt), labelled to contain 40 mg PAN.

### 2.2. Reagents and solvents

Double-distilled water was used to prepare all solutions during entire analysis. Methanol was purchased from Fisher Scientific Limited, UK. Uric acid, dopamine and BCG were purchased from Sigma-Aldrich, Germany. Glacial acetic acid, phosphoric acid, boric acid and ascorbic acid, ferric chloride, nickel sulfate, copper sulfate, chromium chloride, zinc sulfate, potassium ferricyanide, potassium chloride were purchased from El Nasr Pharmaceutical Chemicals Co., Egypt).

Britton–Robinson buffer (B.R.) as a supporting electrolyte (equal volumes of 0.04 M acetic acid, 0.04 M phosphoric acid and 0.04 M acetic acid, adjusted to a desired pH by 2 N NaOH).

### 2.3. Instrumentation

A Princeton VersaSTAT MC (VersaSTAT 3, Model RE-1, Princeton Applied Research, AMETEK, USA) connected to a three-electrode cell was used for the electrochemical measurements. In all measurements, the reference electrode was Ag/AgCl (3 M KCl), the auxiliary electrode was a platinum wire and PGE the working electrode. A Pentel pencil, Model P205 (Japan), was used as a holder for the pencil lead. Electrical contact with the lead was achieved by soldering a metallic wire to the metallic part that holds the lead in place inside the pencil. Unless stated otherwise, the pencil was fixed so that about 3 mm of its length is immersed into the solution. Measurements were performed in a 10 ml glass cell containing 6 ml of supporting electrolyte solution. Stirring was achieved with a magnetic stirring bar. The pH values of solutions were measured using Hanna pH meter (Hanna Instruments Brazil, São Paulo Brazil) with a combined electrode. The solutions were sonicated using Bransonic ultrasonic cleaner Branson UL Transonics Corporation Danbury, USA. Surface morphology studies of the modified electrode were carried out using a scanning electron microscope (SEM) JEOL JSM-5400 LV instrument (Oxford, USA). A Nicolet 6700 FTIR advanced Gold Spectrometer supported with OMNIC 8 software (Thermo Electron Scientific Instruments Corp. WI, USA) was used for data processing.

### 2.4. Preparation of standard solutions

An accurately weighed amount of PAN sodium was transferred into a 100 ml calibrated flask and dissolved in about 10 ml methanol. The solution was completed to the mark with distilled water to provide a stock solution containing 1.0 mM of PAN sodium. The working standard solutions were prepared by further dilution of the suitable aliquots of the stock solution with B.R. buffer (pH = 7.0).

### 2.5. Real sample preparation

The contents of 10 tablets were accurately weighed, finely powdered and thoroughly mixed in a mortar. Portions equivalent to about 1.0 mM of each drug was accurately weighed and dissolved in 20 ml methanol. The contents were sonicated for 20 min to assure complete solubility. The excipients were separated by centrifugation at 3000 r.p.m. for 5 min. The residue was washed three times with distilled water.

For vials, accurate weighed amount of the powder equivalent to about 1.0 mM for PAN sodium was dissolved in methanol shaken well for 5 min and sonicated for further 5 min. The filtrate was transferred into a 100 ml calibrated flask and diluted to a final volume with distilled water. Appropriate working solutions were prepared by taking suitable aliquots from these stock solutions and diluting them with the B.R. buffer solutions (pH = 7.0).

Drug-free rabbit plasma samples were obtained from six healthy rabbits stored at −20°C and analysed the next day after collection without any further pretreatment. One millilitre of the plasma was pipetted into a voltammetric cell containing 6 ml B.R. buffer (pH = 7.0).

### 2.6. Procedure for application *in vivo* (pharmacokinetic study)

The pharmacokinetic of PAN (Pantazol^®^ injection) was evaluated in plasma using six healthy male rabbits (1800–2200 g) following a single intra-peritoneal injection (I.P.). Blood samples were collected pre-dosing and at 10, 15, 30 min and 1, 2, 4, 8, 12 h (s) after the I.P. administration into heparinized tubes. The blood samples were centrifuged immediately at 3000 r.p.m. for 15 min, and then plasma fractions were rapidly separated and stored in cooled polypropylene tubes at −20°C. The plasma samples were analysed using the described square wave adsorptive stripping voltammetric (SWAdSV).

## 3. Results and discussions

### 3.1. Deposition of poly (bromocresol green) film on the pencil graphite electrode surface and parameters affecting deposition

#### 3.1.1. Preparation of poly (bromocresol green)/pencil graphite electrode

**Figure 1.**
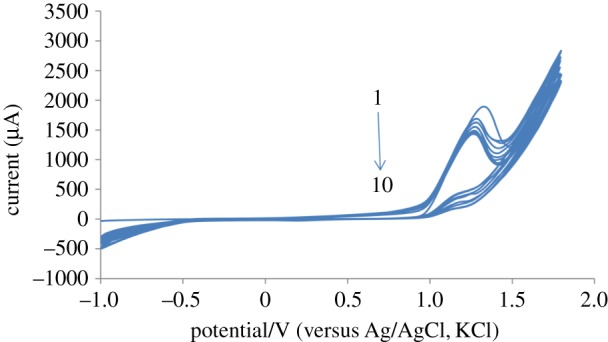
Successive cyclic voltammograms (10 cycles) for electro-polymerization of BCG on PGE surface. Conditions were 0.4 mM of BCG in 0.1 M, NaOH, scan rate of 100 mV s^−1^.

**Scheme 1.**
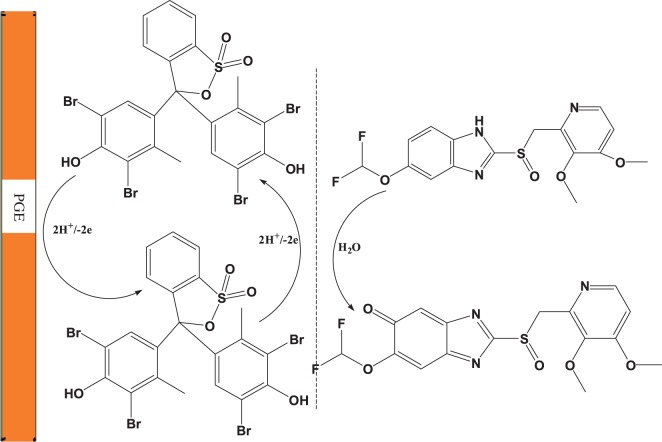
Proposed electro-catalytic mechanism for the oxidation of PAN sodium on the surface of poly (BCG)/PGE.

Figure 1 shows the successive cyclic voltammograms recorded during the electro-polymerization of BCG on the PGE surface in NaOH solution (0.1 M). The results have been shown that the oxidation peak for BCG has gradually decreased during the successive multi-cycles. This could confirm deposition of the poly (BCG) film on the PGE surface according to the proposed electro-polymerization process (scheme 1).

#### 3.1.2. Study of poly (bromocresol green) film on pencil graphite electrode

**Figure 2.**
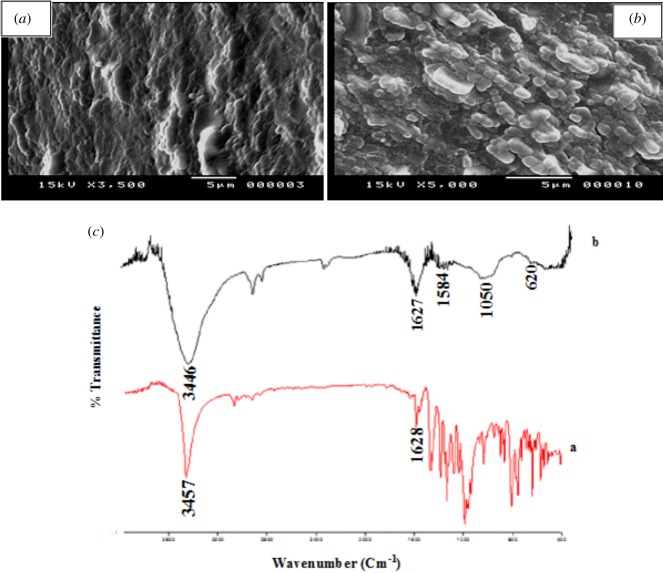
SEM of bare PGE (*a*) and BCG/PGE (*b*) and (*c*) FTIR spectra of BCG (a) and poly (BCG) film (b).

Scanning electron microscopy was used to investigate the interface morphology of the electrode surfaces. Figure 2*a* and *b* shows the surface morphology of bare PGE and poly (BCG)/PGE, respectively. A smooth surface was clearly observed on the bare PGE. However, after electro-polymerization of BCG the surface of PGE was covered with a uniform flake shaped film indicating that the BCG film was successively modified on the electrode surface. FTIR spectroscopy has been also used to further confirm modification of the electrode (figure 2*c*). The close inspection of the spectra has revealed that the characteristic υ (C=C), υ (C–H) and υ (OH) bands at 1628, 2923 and 3457 cm^−1^, respectively, of the bare electrode were clearly down shifted to 1627, 2890 and 3446 cm^−1^ in the polymerized electrode. These shifts clearly further confirm the polymerization process.

#### 3.1.3. The effect of bromocresol green concentration and deposition cycles

**Figure 3.**
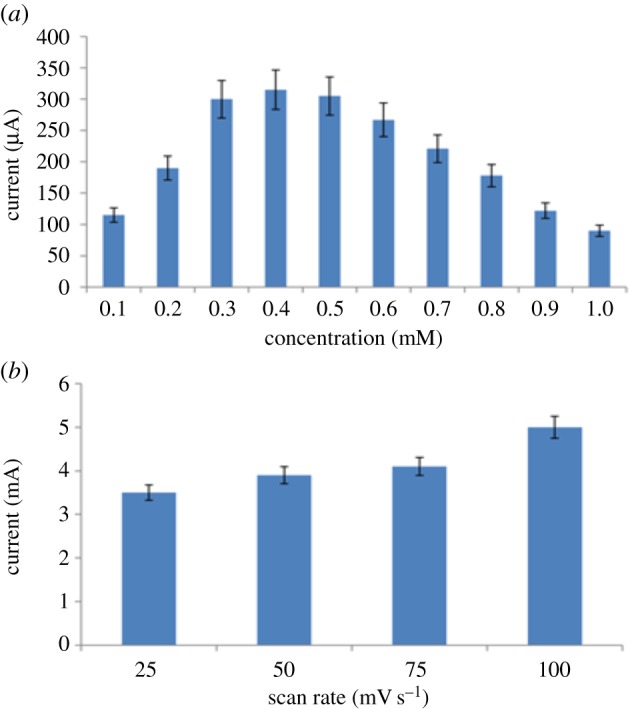
(*a*) The effect of BCG concentration on anodic current of PAN sodium. (*b*) Effect of scan rate on BCG deposition.

The peak current of both PAN sodium was found to increase with the increase of BCG deposition that, in turn, increased with the increase of BCG concentration until 0.4 mM BCG that was taken as an optimum value during the electro-deposition of the BCG on PGE for subsequent analysis of the cited drug (figure 3*a*). After this concentration, the peak current of PAN sodium decreased, this may be attributed to blocking of PGE surface by excess BGC. The maximum currents were achieved after the cycles increase up to 10 cycles, after that the current was decreased that may be attributed to blocking PGE surface by increasing amount of BCG.

#### 3.1.4. The effect of initial deposition potential and time on bromocresol green deposition

The effect of adsorption potential and time on the BCG through measuring the anodic peak current of PAN sodium was studied at various adsorption potentials between –1.2 V and +2.0 V. The maximum peak current was obtained at an adsorption potential of −1.0 V and after 5 s.

#### 3.1.5. The effect of scan rate on deposition bromocresol green at electrode surface

The effect of scan rate on the redox behaviour of the deposited poly (BCG) film was investigated in the scan rate range of 25–100 mV s^−1^. The cyclic voltammograms of the poly (BCG) film recorded at different scan rates are shown in figure 3*b*.

### 3.2. Electrochemical characterization of modified electrode using standard potassium ferricyanide

**Figure 4.**
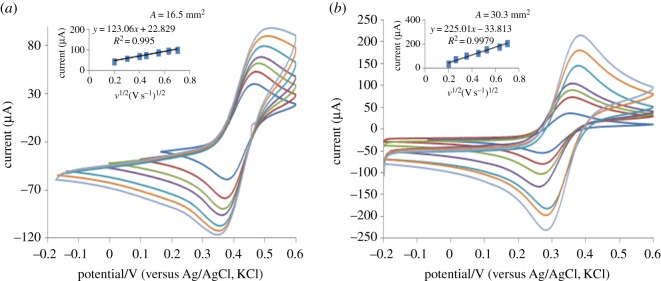
The electro-activity of bare PGE (*a*) versus poly (BCG)/PGE (*b*) using CV and 1 mM potassium ferricyanide in 0.5 M KCl at scan rate 100 mV s^−1^.

Prior to voltammetric analysis the PGE was evaluated. The cyclic voltammetry (CV) was recorded on PGE wetted with 0.5 M KCl where no voltammetric peaks were recorded. Thus no electro-active interfering species were appreciably released by all graphite sticks. Furthermore, the CV was recorded again after wetting PGE with 1 mM potassium ferricyanide in 0.5 M KCl. The Randles–Sevick equation for a reversible process [38] was used to estimate effective surface area of the PGE (*A*_eff. _mm^2^) immersed in 1 mM potassium ferricyanide and 0.5 M KCl:

$$I_{\mathrm{pa}}=(2.69\times 10^{5})n^{2/3}A_{\mathrm{eff}.}D^{1/2}v^{1/2}C^{\circ },$$

where *D* and *C*° are the diffusion coefficient and bulk concentration of the redox probe, respectively. The electro-active surface area of the bare PGE was 16.5 mm^2^ (figure 4*a*) while that of the poly (BCG)/PGE was 30.3 mm^2^ (figure 4*b*). Compared with the bare PGE the electro-active surface area of the modified PGE increased approximately 83.6%.

### 3.3. Electrochemical characterization of pantoprazole sodium at bare electrode versus modified electrode

**Figure 5.**
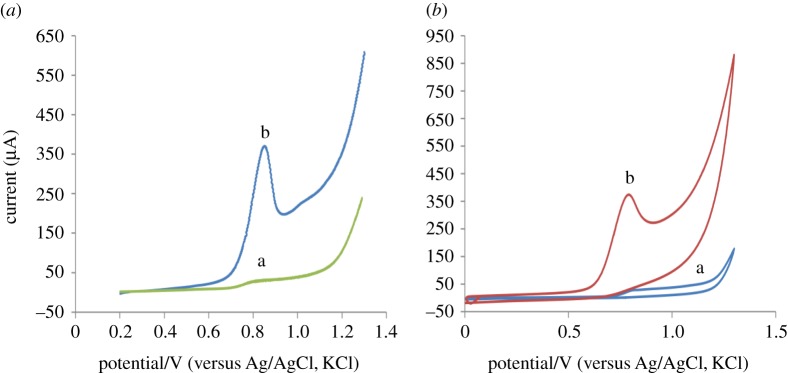
The SWAdSV (*a*) and CV (*b*) at bare PGE (a) and poly (BCG)/PGE (b).

The CV and SWAdSV were presented in figure 5*a* and *b*, respectively. The CV of PAN sodium displayed only a single irreversible anodic peak at +0.83 V and no cathodic peak in the reverse scan was recorded which means that the oxidation of PAN sodium is irreversible.

### 3.4. Analytical parameters involved in the square wave adsorptive stripping voltammetric determination of pantoprazole sodium

#### 3.4.1. The effect of pH

**Figure 6.**
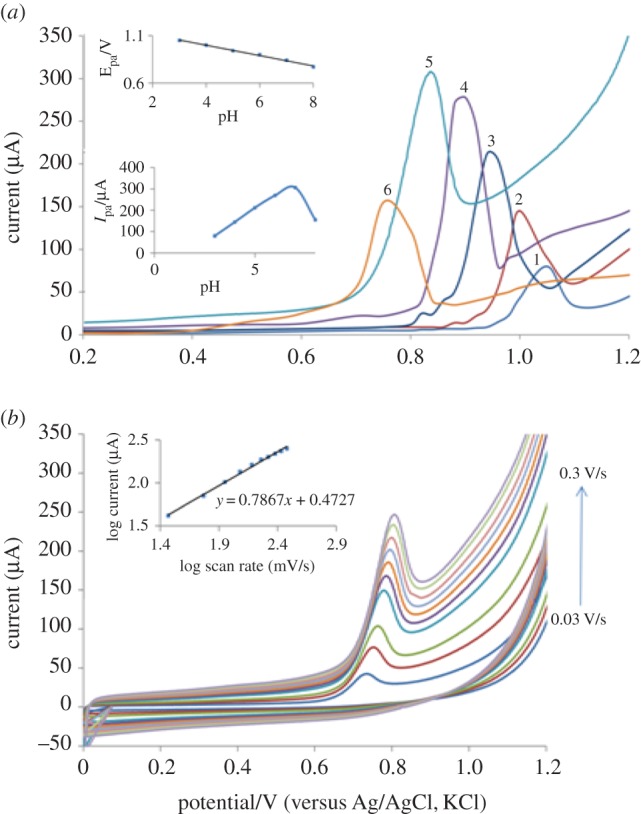
(*a*) The effect of pH on SWAdSV of PAN sodium: (1) pH = 3.0 (2) pH = 4.0 (3) pH = 5.0 (4) pH = 6.0 (5) pH = 7.0 and (6) pH = 8.0; and (*b*) the effect of scan rate on CV of PAN sodium.

Electro-oxidation of PAN sodium was studied with different supporting electrolytes such as B.R. buffer acetate and phosphate buffer by CV. The B.R. buffer produces better results as compared to the other supporting electrolytes. Hence B.R. buffer was chosen as a supporting electrolyte. Well-defined sharp oxidation peaks were recorded at pH = 7.0. The peak potential (*E*_pa_) shifted towards less positive side with increasing pH. The optimum result with respect to sensitivity accompanied with sharper response was obtained at pH = 7.0 (figure 6*a*).

#### 3.4.2. The effects of initial deposition potential and time

**Figure 7.**
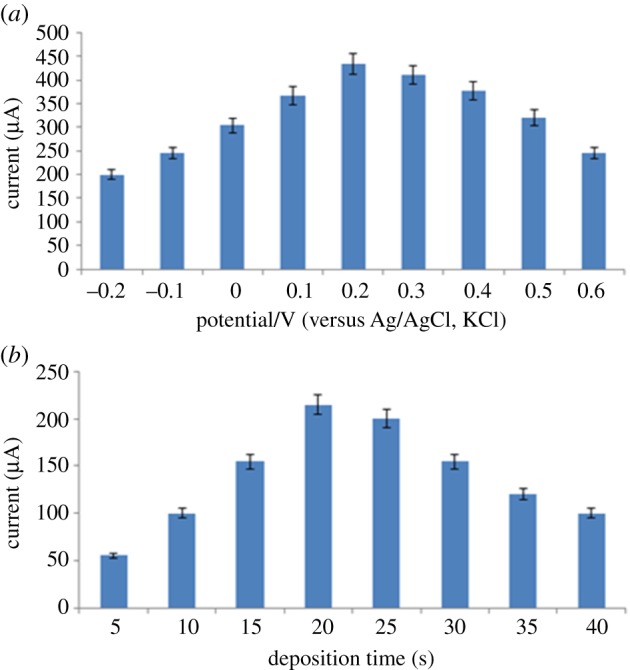
The effect of adsorption potential (*a*) and adsorption time (*b*) on oxidation of PAN sodium.

The effect of adsorption potential and time on the anodic peak current of PAN sodium was studied using poly (BCG) electrode at various adsorption potentials between −0.2 V and +0.6 V for SWAdSV in B.R. buffer (pH 7.0). The maximum peak current was obtained at an adsorption potential of 0.2 V and after 20 s (figure 7).

#### 3.4.3. Effect of scan rate

Figure 6*b* shows the effect of scan rate in the range of 30–150 mV s^−1^ on the CV response of PAN sodium in B.R. buffer (pH 7.0). With increasing scan rates, the anodic peak slightly shifted to the positive potential side. The peak current is increasing remarkably with increasing scan rates. From the value of the slope, it can be deduced that the electrochemical oxidation process of PAN sodium is a diffusion-controlled process with an adsorption contribution. For an adsorption-controlled electrode reaction, the following equation could apply [39]:

$$I_{\mathrm{pa}}=\frac{nFQv}{4RT},$$

where *Q* is the peak area that could be obtained under given scan rate; *v* is the scan rate; *F*, *R* and *T* are constants. From the slope of *Ip* versus *ν* the electron-transfer number (*n*) that was involved in the electrode reaction of PAN sodium was calculated to be 2.

## 4. Method validation

The optimum conditions for the determination of PAN sodium using SWAdSV were: adsorption potential = 0.2 V, frequency = 180 Hz, step potential = 20 mV and potential amplitude = 55 mV. Under these optimum conditions, a linear calibration plot was recorded.

### 4.1. Linearity limit of detection and limit of quantitation

**Figure 8.**
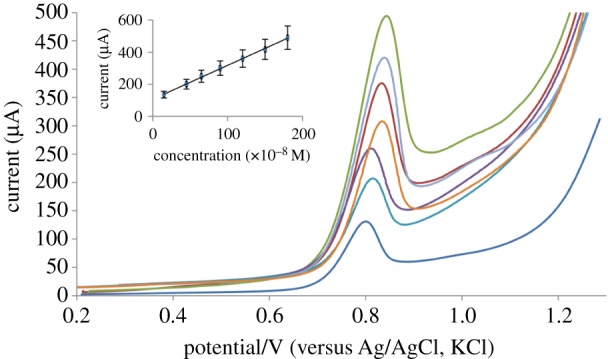
The effect of concentration on SWAdSV of PAN sodium (15–180 × 10^−8^ M). Conditions were: frequency = 180 Hz step potential = 20 mV and potential amplitude = 55 mV.

Figure 8 has shown a linear calibration plot over the concentration range under the optimum conditions. Table 1 shows good values of the correlation coefficient (*r*) with small intercept, small value of standard deviation (S.D.) and relative standard deviation (RSD) that point out high accuracy and precision of the proposed method. The low values of limit of detection (LOD) and limit of quantitation (LOQ) reflect that SWAdSV is sensitive for PAN sodium determination. The LOD and LOQ were calculated by using the following equations [40]: LOD = 3.3 s/S and LOQ = 10 s/S where ‘s’ is the mean of standard deviation of intercept and ‘S’ is the mean of the slope of the calibration curve. The LOD and LOQ are presented in table 1. Obviously, the low values of LOD and LOQ would indicate that the SWAdSV is highly sensitive for the determination of PAN sodium.

**Table 1.** The quantitative parameters of the proposed SWAdSV method.

analytical parameters	values
linearity range (×10^−8^ M)	40–350
correlation coefficient	0.9994
intercept (a)± s.d.^a^	70 ± 1
slope (b)± s.d.^a^	1.5 ± 0.02
LOD^a^(×10^−8^ M)	2.2
LOQ^a^(×10^−8^ M)	6.6

^a^Average of five replicates.

### 4.2. Accuracy

The accuracy of the method was determined by adding known amounts of PAN sodium to sample solution (tablets and vials) and calculating the recovery percentages. The results have indicated good accuracy of the proposed method (tables 2 and 3). These results have also proved the absence of the interference owing to common excipients.

**Table 2.** The assay of PAN sodium in their pharmaceutical dosage forms by the proposed voltammetric method.

		proposed method	reported method			
studied drug	pharmaceutical formulation	% recovery ± s.d.^a^		Student's *t*-test	*F*-test	reference
PAN sodium	Pantoloc^®^ tablets	100.1 ± 1.7	99.1 ± 1.4	2.3	5.4	[10]
	Pantazol^®^ vials	101.0 ± 1.4	99.3 ± 1.4	2.7	5.1	

^a^Average of five replicates.

**Table 3.** The standard addition method for determination of PAN in pharmaceutical formulation using the proposed method.

drug	pharmaceutical formulation	amount taken (×10^−8^ M)	amount added (×10^−8^ M)	total amount found (×10^−8^ M)	% recovery ± S.D.^a^
PAN sodium	Pantoloc^®^ tablets	20	20	39.3	98.3 ± 1.5
			40	58.7	97.8 ± 1.1
			60	81.5	101.8 ± 1.7
	Pantazol^®^ vials	20	20	40.5	101.3 ± 1.0
			40	60.9	102.3 ± 1.3
			60	79.0	98.3 ± 1.2

^a^Average of five replicates.

### 4.3. Precision

The results of the inter-day and intra-day precision of the proposed method were ranged from 98.0 to 100.8% ± (1.2–1.7). The inter-day and intra-day precisions were evaluated through replicate analysis of the studied drug. The precision of the proposed methods was fairly high as indicated by the low values of S.D. and %RSD.

### 4.4. Selectivity of the method

The effects of common excipients co-administered drugs biologically active compounds and divalent metals were evaluated (table 4). Clearly, the % signal change of 120 × 10^−8^ M PAN sodium upon addition of these potential interfering substances has not changed appreciably. This could indicate the selectivity of the method and hence its suitability for the determination of pharmaceuticals in complex matrices.

**Table 4.** The influence of potential components on the voltammetric response of PAN sodium.

common excipients		biological active substances		co-administered drugs		cations	
amount (1 mM)	%signal change^a^	amount (1 mM)	%signal change^a^	amount (20 µM)	%signal change^a^	amount (0.3 µM)	%signal change^a^
starch	3.55	ascorbic acid	4.23	domperidone	4.34	manganese	2.80
glucose	2.22	uric acid	4.10	aceclofenac	3.22	nickel	3.23
gum acacia	3.45	dopamine	3.32	metronidazole	0.09	copper	8.97
lactose	3.22			clarithromycine	1.98	cadmium	6.32
citric acid	4.34			doxycycline	2.21	zinc	5.43
						chromium	6.45
						calcium	2.13
						magnesium	1.22
						potassium	1.01
						sodium	0.87

^a^Average of five replicates.

### 4.5. Application to pharmaceutical dosage forms

The proposed method was applied to the determination of PAN sodium in tablets and vials (table 2). The results were compared with the reported method [10]. The results of the proposed method were found to be comparable with those of the reported method as indicated by *t*- and *F*-tests. In addition, recovery studies were performed by using a standard addition method.

### 4.6. *In vitro* and *in vivo* studies

#### 4.6.1. Validation of the proposed method in rabbit plasma

##### 4.6.1.1. Linearity

The linearity of the proposed method in rabbit plasma was 55–330 × 10^−7^ M with a correlation coefficient of 0.9989 ± 0.009 (table 5).

**Table 5.** Pharmacokinetic parameters of PAN sodium using SWAdSV.

pharmacokinetic parameters^a^	SWAdSV
*C*_max_	31
*t*_max_/min	30.0
*t*_1/2_ (h) = 0.693/*K*_el_	6.1
MRT_0–12_ (M h)	4.5
MRT_0–∞_ (M h)	9.3
AUC_0–12_(M h)	167.6
AUC_0–∞_ (M h)	233.3
*K*_el_ (1/h)	0.13

^a^Parameters are *C*_max_, maximum plasma concentration; *t*_max_, maximum plasma concentration; *t*_1/2_, elimination half-life of drug; MRT_0–12_, mean residual time up to 12 h; MRT_0–∞_, mean residual time up to infinity; AUC_0–12_, area under the curve up to 12 h; AUC_0–∞_, area under the curve up to infinity; *K*_el_, apparent terminal rate constant; AUMC_0−*t*_, the area under the first moment; CL, clearance.

##### 4.6.1.2. Accuracy

The accuracy of the proposed method was determined by investigating the recovery percentages of PAN sodium at three concentration levels covering low medium and high range of the calibration curve. The results of 100.2–103.7% (±1.4–2.1) revealed good accuracy of the proposed method.

##### 4.6.1.3. Precision

The precision was evaluated by measuring intra-day and inter-day precision. The intra-day values were calculated at three concentration levels in the same day with good coefficient of variation values. The inter-day precision was evaluated at three different days with good values. The results of 97.8–99.3% (± 0.9–1.6) show good repeatability and reproducibility of the proposed method in rabbit plasma.

#### 4.6.2. Pharmacokinetic study

**Figure 9.**
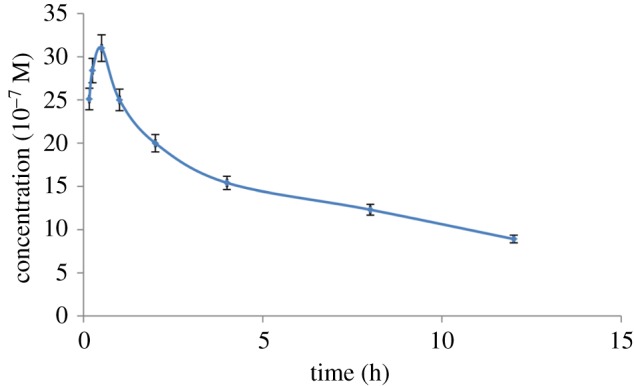
Mean plasma concentration–time profiles for six male rabbits following an intraperitoneal administration of a single dose of pantazol^®^ (0.55 mg kg^−1^). Conditions were: frequency = 180 Hz step potential = 20 mV and potential amplitude = 55 mV.

Owing to high sensitivity of the proposed SWAdSV method as expressed by low values of LOD and LOQ, the method was applied for the pharmacokinetic study of PAN sodium in rabbit plasma for, to our knowledge, the first time. The concentration–time curve in rabbits following the I.P. administration of pantazol^®^ injection containing PAN sodium at a dose of 0.55 mg kg^−1^ (*n* = 6) is shown in figure 9. The drug concentration in rabbit plasma decreased gradually (*T*_max = _30 min) after an I.P. injection in this study. The pharmacokinetic parameters were estimated by the non-compartmental analysis using moment analysis Microsoft Excel 2003. The relevant pharmacokinetic parameters are shown in table 6.

**Table 6.** Comparison of the proposed method with reported electrochemical methods for analysis of PAN sodium.

technique(s)	electrode	linear range (µM)	LOD (µM)	application	reference
DPV	glassy carbon	6–800	0.4	tablets and human plasma	[10]
SWAdSV	*ex situ* plated antimony-film	9–200	0.9	tablets	[11]
DPV and SWAdSV	glassy carbon	0.5–7.5 and 0.6–4.3, respectively	0.03 and 0.007, respectively	tablets and human urine	[12]
DPV	carbon paste	0.1–10	0.02	tablets	[13]
SWAdSV	hanging dropping mercury electrode	1000–5000	---	tablets	[15]
SWAdSV	hanging dropping mercury electrode	0.15–25.23	0.048	tablets, spiked plasma	[16]
SWAdSV	poly (BGE)/PGE	0.066–3.6	0.022	tablets, vials and rabbit plasma	this study

## 5. Reaction mechanism

The oxidation mechanism proposed scheme 1 is based on the electrochemical data described and supported by the PAN sodium structure with two systems that are in resonance separated by a methylene group. The oxidation step in the ortho position will be favoured via resonance by the stabilization of the radical formed. Preferential attack is in the aromatic ring where the extent of conjugation is greater making removal of an electron easier. One electron is removed followed by deprotonation to produce a cation radical which reacts with water and leads to the formation of quinone species [41].

## 6. Comparison of the proposed method with other reported methods

Table 6 summarizes the analytical performances of our proposed and the literature methods in terms of linearity LOQ and LOD. Our method has the lowest LOD for PAN sodium compared with the literature methods. These results could indicate that the poly (BCG)/PGE method would be an attractive alternative choice for determining of the cited drug in pure form pharmaceutical formulations and biological fluids.

## 7. Conclusion

In summary, a simple and fast electrochemical method was used for determination of PAN sodium by electro-polymerization of BCG on pencil graphite electrode. It showed excellent electro-catalytic activity towards the oxidation of the studied drug. The sensor exhibited wide linear range, low detection limit and high selectivity. Moreover, it was used for determination of PAN sodium in pharmaceutical formulations and during pharmacokinetic studies. Therefore, the proposed method provides a promising platform for determination of the cited drug in the field of electro-analytical chemistry.

## Data accessibility

This article has no additional data.
